# A Case Report of Pericardial Constriction and Left Ventricular Dysfunction in a Breast Cancer Survivor: Late Cardiotoxicity From Radiotherapy or 5-Fluorouracil

**DOI:** 10.7759/cureus.83682

**Published:** 2025-05-07

**Authors:** Taha Berhil, Fadoua Lahnaoui, Badre El Boussaadani, Amine Ech-Chenbouli, Zainab Raissouni

**Affiliations:** 1 Cardiology, Mohammed VI University Hospital Center, Tangier, MAR; 2 Cardiology, Abdelmalek Essaadi University, Tangier, MAR

**Keywords:** 5-fu chemotherapy, cardiotoxicity, constrictive pericarditis, left ventricular dysfunction, long-term cardiovascular monitoring, radiotherapy-induced cardiac injury

## Abstract

Cardiotoxicity from oncologic treatments, including radiotherapy and fluoropyrimidine-based chemotherapy, can manifest years later, leading to pericardial constriction and left ventricular dysfunction in cancer survivors.

We report the case of a 53-year-old breast cancer survivor, treated with surgery, radiotherapy (>25 Gy), and 5-fluorouracil (5-FU) chemotherapy in 2015, who remained cancer-free until a pleural recurrence in 2024, managed with pleurodesis and capecitabine. Six months later, she developed cardiac tamponade requiring pericardial drainage. Transthoracic echocardiography showed a preserved left ventricular ejection fraction (LVEF) (60%), and concurrent subclavian vein thrombosis led to anticoagulation. By early 2025, she developed left ventricular dysfunction (LVEF 45%) with exertional dyspnea classified as New York Heart Association (NYHA) class III. NT-proBNP levels were elevated. Coronary disease was excluded. Cardiac magnetic resonance imaging (MRI) revealed evolving constrictive pericarditis, moderate dysfunction (global longitudinal strain (GLS)-13.5%), biatrial enlargement, and bilateral pleural effusion. Right heart catheterization confirmed adiastole with a deep plateau pattern.

This case highlights a rare late-onset pericardial constriction and ventricular dysfunction in a breast cancer survivor, potentially linked to prior oncologic treatments. These findings underscore the importance of long-term cardiovascular monitoring in cancer survivors.

## Introduction

Advancements in cancer treatment have significantly improved survival rates, leading to a growing population of long-term cancer survivors. However, this positive trend has brought increasing attention to the long-term complications of cancer therapy, particularly cardiovascular disease (CVD), which has emerged as a leading cause of non-cancer-related morbidity and mortality in this group. Studies suggest that cancer survivors have a 1.5 to three times higher risk of developing cardiovascular complications compared to the general population, depending on the type of cancer, treatment received, and individual risk factors [1].

Cardiotoxicity is a well-recognized adverse effect of several cancer therapies, including radiotherapy and chemotherapeutic agents such as 5-fluorouracil (5-FU). The incidence of cardiotoxicity varies from 5% to 20%, with some patients developing acute manifestations and others experiencing delayed structural damage [2]. Radiotherapy, especially when involving the thoracic region, has been associated with long-term effects such as pericardial fibrosis and constrictive pericarditis, often with a latency of 10 years or more [3]. Conversely, 5-FU is more commonly linked to acute coronary vasospasm and myocardial ischemia [4].

Despite the relatively low prevalence of pericardial constriction, affecting fewer than 2% of breast cancer survivors, it represents a clinically significant and potentially underdiagnosed complication due to its insidious onset and diagnostic complexity [3]. In this context, our case highlights the importance of considering overlapping cardiotoxic mechanisms in patients with a history of multimodal cancer therapy. It also underscores the need for long-term cardiovascular surveillance as an integral part of oncologic follow-up, even in asymptomatic survivors.

## Case presentation

We report the case of a 53-year-old postmenopausal female with a history of left breast cancer diagnosed in 2015. The patient was treated with surgical resection, followed by adjuvant radiotherapy to the left chest wall and regional lymphatics (50 Gy in 25 fractions over five weeks), and FU-based chemotherapy (5-FU). She achieved complete remission and was declared cancer-free before 2024.

In early 2024, she presented with a pleural recurrence confirmed on histopathology, which was managed by left-sided pleurodesis. She was initiated on capecitabine. Six months later, in October 2024, she was hospitalized for cardiac tamponade, necessitating pericardial drainage of two liters of hemorrhagic fluid. However, cytological analysis of the pericardial fluid was negative for neoplastic cells, making malignancy less likely, though not definitively excluded; a pericardial biopsy may have offered greater diagnostic yield but was not performed.

During this admission, echocardiography revealed a preserved left ventricular ejection fraction (LVEF) (60%). Additionally, a right subclavian vein thrombosis was diagnosed, and the patient was started on apixaban. Given her cardiac involvement, capecitabine was discontinued.

In January 2025, follow-up imaging revealed newly developed left ventricular dysfunction with a moderately reduced ejection fraction (45%). At the time of presentation, the patient reported persistent exertional dyspnea classified as New York Heart Association (NYHA) class III. Clinical examination was unremarkable. The troponins were negative, and the NT-proBNP levels were elevated at 2500 pg/mL, with an upper limit of 400 pg/mL. The inflammatory workup and renal function tests were normal. The electrocardiogram showed a sinus rhythm without conduction or rhythm disturbances, an isoelectric ST segment, normal voltage, and no atrial enlargement.

**Figure 1:**
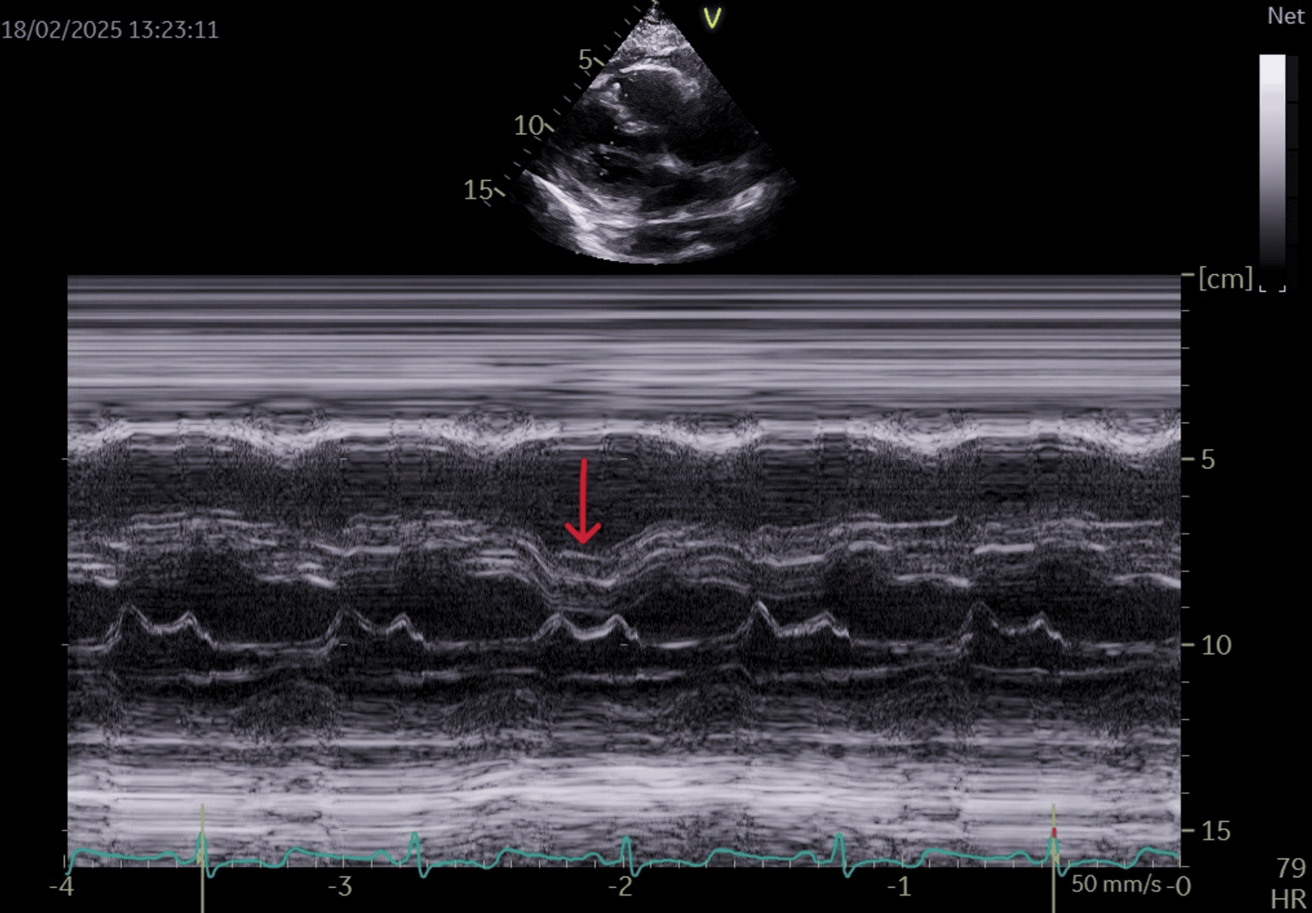
An M-mode echocardiographic image in the parasternal long-axis view shows diastolic septal bounce (arrow), with paradoxical motion of the interventricular septum toward the left ventricle during inspiration, a sign of right-left ventricular interdependence

**Figure 2:**
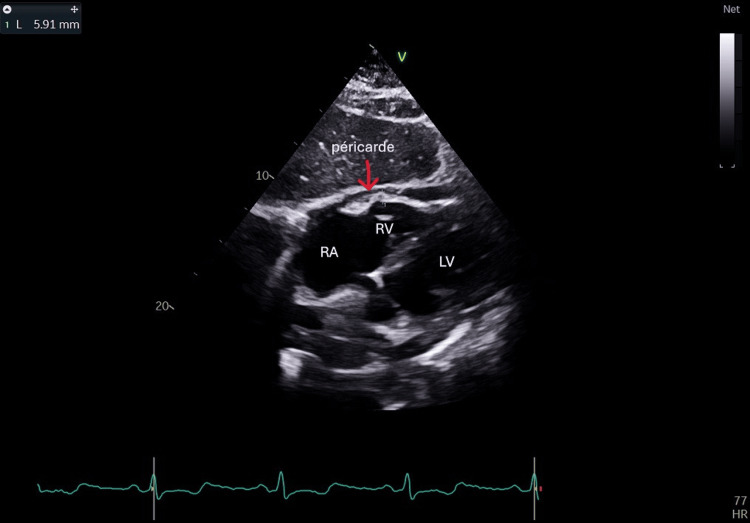
A subcostal four-chamber view showing pericardial thickening measuring 6 mm (normal ≤4 mm) (arrow), which is a characteristic finding in constrictive pericarditis LA: left atrium; LV: left ventricle; RA: right atrium

**Figure 3:**
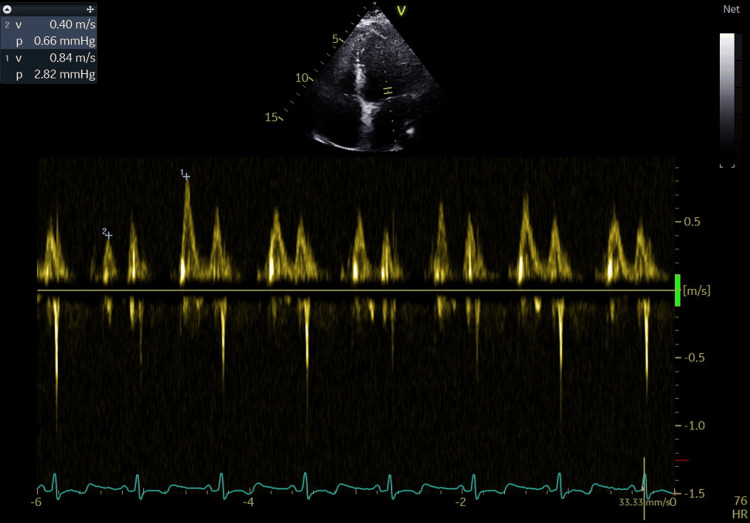
Pulsed Doppler mitral inflow shows 55% respiratory variation, exceeding the ≥25% threshold, suggestive of constrictive pericarditis. The variation reflects enhanced ventricular interdependence, with decreased left ventricular filling during inspiration and increased filling during expiration due to the non-compliant pericardium

Coronary angiography (January 30, 2025) excluded significant coronary artery disease. Echocardiography reveals multiple findings suggestive of chronic constrictive pericarditis (CCP), including septal bounce (Figure 1), pericardial thickening (Figure 2), respiratory variation in mitral and tricuspid inflows (Figure 3), and diastolic reversal of the D-wave in the hepatic vein flow.

**Figure 4:**
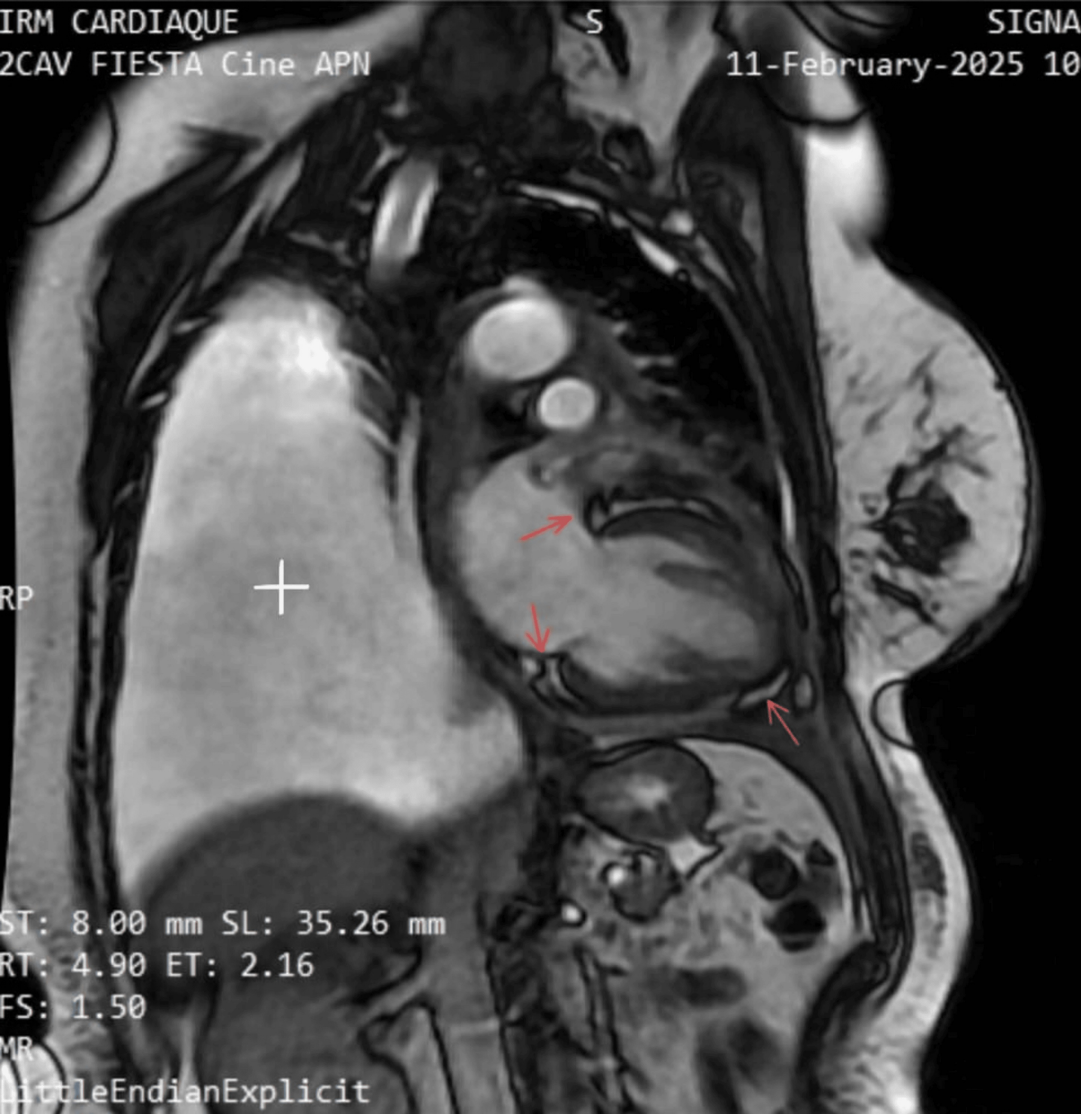
Cardiac MRI in a cine two-chamber view shows subacute inflammatory pericarditis (red arrows) with an evolving constrictive pattern affecting ventricular filling and a large pleural effusion (white plus) MRI: magnetic resonance imaging

Cardiac MRI (Figure 4) demonstrated findings consistent with subacute inflammatory pericarditis with evolving constrictive physiology. Notable findings included moderate cardiac dysfunction (GLS at -13.5% with a normal reference value set at -20%) and biatrial enlargement (left atrium: 26 cm² (normal <21 cm²), right atrium: 20 cm² (normal <18 cm²)). It did not show late gadolinium enhancement, no myocardial infarction sequelae, myocarditis, interstitial fibrosis, or intracavitary thrombus were identified. Furthermore, a bilateral pleural effusion with an inflammatory profile was noted, predominantly on the left side.

**Figure 5:**
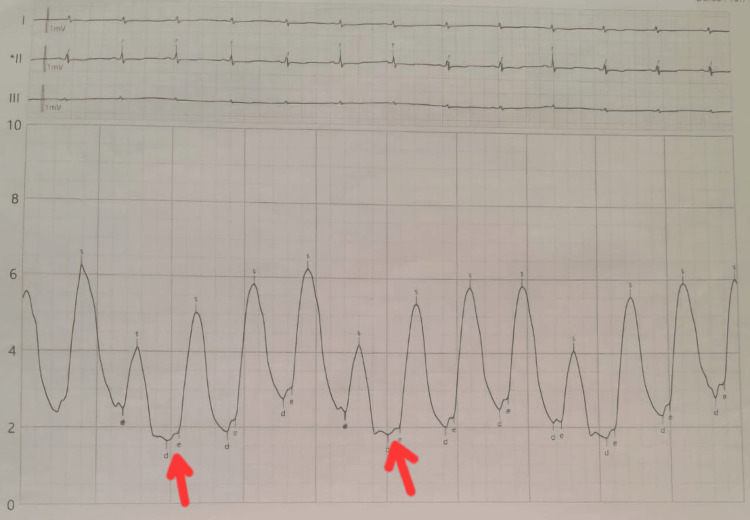
Right heart catheterization shows a typical "dip-and-plateau" (square root) pattern. The red arrows highlight the rapid early diastolic dip followed by a plateau, which is characteristic of constrictive pericarditis

The right catheter shows a deep plateau appearance confirming adiastole (Figure 5).

Although other causes such as infection, autoimmune disease, or malignancy-related constriction were considered, investigations (negative serology, cytology, and imaging) helped exclude these possibilities.

The patient was treated with diuretics, direct oral anticoagulants (DOACs), and a cardioprotective treatment based on beta-blocker, specifically carvedilol, was initiated at a dose of 6.25 mg twice daily, along with ramipril at a dose of 5 mg, with subsequent treatment titration. Pericardectomy was proposed, but the patient refused the surgery.

Three months later, during follow-up, she maintained a moderately reduced LVEF without signs of decompensated heart failure and a stable NYHA class II dyspnea. In this context, the optimization and titration of medical therapy are particularly crucial, serving as the cornerstone of management when surgical intervention is not pursued.

## Discussion

This case illustrates a potential delayed onset of cardiotoxicity secondary to oncologic therapies, manifesting as pericardial constriction and left ventricular dysfunction in the absence of coronary artery disease. Two major etiologies are considered: radiation-induced cardiotoxicity and fluoropyrimidine-associated cardiac toxicity.

Radiation therapy is well recognized as a cause of late-onset cardiovascular complications, particularly when doses exceed 25 Gy, as was the case in this patient. Radiation-related pericardial involvement may present as pericardial effusion, chronic pericarditis, or progressive pericardial fibrosis leading to constrictive pericarditis [4,5]. The latency period for radiation-induced pericardial constriction may range from several years to over a decade, making this a plausible explanation for the current presentation [1]. Furthermore, radiation therapy is associated with left ventricular dysfunction due to myocardial fibrosis, microvascular damage, and diastolic impairment [6]. The absence of significant coronary artery disease on angiography reinforces the hypothesis of a non-ischemic origin of the left ventricular dysfunction, which aligns with a radiation-induced fibrotic process affecting the myocardium.

5-FU and its derivatives, including capecitabine, are known to cause acute cardiotoxic effects, predominantly in the form of coronary vasospasm, myocardial ischemia, or myocarditis [7]. Although less frequently reported, fluoropyrimidine-induced pericarditis and pericardial fibrosis have been documented [8,9].

Similarly, left ventricular dysfunction in patients exposed to 5-FU has been described even in the absence of obstructive coronary artery disease, suggesting an alternative pathophysiological mechanism such as microvascular dysfunction, direct myocardial toxicity, or inflammatory changes [10]. Given the temporal association with her chemotherapy exposure, it remains conceivable that 5-FU contributed to both the pericardial involvement and left ventricular dysfunction, albeit as a rarer complication.

It is highly plausible that the cardiac pathology in this patient is the result of a combination of radiotherapy and chemotherapy-related damage. Radiation therapy is the most likely etiology for pericardial fibrosis and left ventricular dysfunction, but 5-FU may have exacerbated myocardial injury through inflammatory or microvascular pathways. The chronology also supports a dual role of therapies: capecitabine-induced pericardial inflammation (noted during active treatment) and delayed radiation-induced myocardial dysfunction. While 5-FU and capecitabine can provoke acute coronary syndromes through coronary vasospasm, capillary leak, and endothelial injury, they may also trigger pericarditis and myocardial inflammation through microvascular toxicity.

The presence of subclavian vein thrombosis also raises the question of a paraneoplastic hypercoagulable state, though its contribution to pericardial or pleural pathology remains speculative.

This case underscores the necessity of long-term cardiovascular surveillance in cancer survivors, particularly those who have received potentially cardiotoxic treatments. Patients with a history of high-dose mediastinal radiation therapy or fluoropyrimidine-based chemotherapy should undergo periodic cardiac imaging and laboratory tests. Long-term surveillance should involve periodic echocardiography, GLS assessment, cardiac MRI if needed, and potential biomarker evaluation (e.g., troponin and NT-proBNP). Early referral to cardio-oncology or advanced imaging could allow for preemptive interventions.

Given the current findings, this patient requires close cardiology follow-up with serial imaging, as well as a multidisciplinary approach to evaluate the need for pericardiectomy if symptomatic constriction progresses. The therapeutic strategy should focus on optimal heart failure management, diuresis for volume control, and consideration of anti-inflammatory therapies in the context of active pericardial disease.

Future research is needed to better define surveillance protocols, validate predictive biomarkers, and develop risk stratification models for late cardiotoxicity.

## Conclusions

This case highlights a clinically significant and increasingly recognized late-onset cardiotoxicity following oncologic therapy in a breast cancer survivor. While once considered rare, such complications are being encountered more frequently due to longer survivorship. The clinical course underscores the diagnostic complexity of differentiating between various etiologies of pericardial and myocardial involvement, namely radiation-induced, chemotherapy-related, and malignancy-associated causes.

Although prior radiation remains the most likely contributor, the temporal association with capecitabine use raises the possibility that fluoropyrimidine-related cardiac injury may have played a triggering or aggravating role in a previously sensitized myocardium. This multifactorial interplay highlights the need for a nuanced diagnostic approach in cardio-oncology.

Given the potential for progressive cardiac impairment, long-term surveillance, and individualized treatment strategies are crucial in optimizing outcomes for cancer survivors exposed to cardiotoxic therapies. Periodic assessment with strain imaging and biomarkers, as well as integration into cardio-oncology follow-up programs, may aid in early detection and risk stratification.

## References

[1] Yusuf SW, Sami S, Daher IN (2011). Radiation-induced heart disease: a clinical update. Cardiol Res Pract.

[2] Darby SC, Ewertz M, McGale P (2013). Risk of ischemic heart disease in women after radiotherapy for breast cancer. N Engl J Med.

[3] Zamorano JL, Lancellotti P, Rodriguez Muñoz D (2016). 2016 ESC position paper on cancer treatments and cardiovascular toxicity developed under the auspices of the ESC Committee for practice guidelines: the task force for cancer treatments and cardiovascular toxicity of the European Society of Cardiology (ESC). Eur Heart J.

[4] Fajardo LF, Stewart JR (1970). Experimental radiation-induced heart disease. I. Light microscopic studies. Am J Pathol.

[5] Heidenreich PA, Kapoor JR (2009). Radiation-induced heart disease: systemic disorders in heart disease. Heart.

[6] Aleman BM, van den Belt-Dusebout AW, De Bruin ML (2007). Late cardiotoxicity after treatment for Hodgkin lymphoma. Blood.

[7] Dyhl-Polk A, Vaage-Nilsen M, Schou M (2020). Incidence and risk markers of 5-fluorouracil and capecitabine cardiotoxicity in patients with colorectal cancer. Acta Oncol.

[8] Sara JD, Kaur J, Khodadadi R (2018). 5-fluorouracil and cardiotoxicity: a review. Ther Adv Med Oncol.

[9] Moslehi JJ (2016). Cardiovascular toxic effects of targeted cancer therapies. N Engl J Med.

[10] Kumar D, Warsha F, Mehta A, Deepak V, Jawad W (2021). 5-fluorouracil induced takotsubo cardiomyopathy complicated by left ventricular thrombosis. Cureus.

